# The immunoglobulin M-degrading enzyme of *Streptococcus suis* (Ide_*Ssuis*_) leads to long-lasting inhibition of the activation of porcine IgM-secreting B cells

**DOI:** 10.1186/s13567-024-01363-1

**Published:** 2024-09-23

**Authors:** Annika Katharina Breitfelder, Wieland Schrödl, Christoph Georg Baums, Gottfried Alber, Uwe Müller

**Affiliations:** 1 Institute of Bacteriology and Mycology, Centre for Infectious Diseases, Faculty of Veterinary Medicine, University of Leipzig, Leipzig, Germany; 2 Institute of Immunology, Centre for Infectious Diseases, Faculty of Veterinary Medicine, BBZ, University of Leipzig, Leipzig, Germany

**Keywords:** *Streptococcus suis*, Ide_*Ssuis*_, IgM, B cell receptor, B cell receptor cleavage, ELISpot

## Abstract

*Streptococcus suis* (*S. suis*) is one of the most important porcine pathogens, causing severe pathologies such as meningitis or polyarthritis. It is also a very successful colonizer of mucosal surfaces. The IgM-degrading enzyme of *S. suis* (Ide_*Ssuis*_) specifically cleaves porcine IgM, which results in complement evasion. On the basis of our previous finding that Ide_*Ssuis*_ also cleaves the IgM B cell receptor in vitro, we verified IgM B cell receptor cleavage ex vivo in whole regional lymph nodes and investigated the working hypothesis that this IgM B cell receptor cleavage results in a long-lasting impaired B cell function. The number of IgM-secreting cells was determined via ELISpot analysis after porcine peripheral blood mononuclear cells had initially been treated with different recombinant *S. suis* proteins and subsequently stimulated with interleukin-2 and the toll-like receptor 7/8 ligand R848. Compared with treatment with medium or recombinant muramidase-released protein, treatment with rIde_*Ssuis*_ but also with a cleavage-deficient variant led to a reduction in the number of IgM-secreting cells as well as the level of secreted IgM. Flow cytometry analysis confirmed that the IgM B cell receptor was cleaved only by rIde_*Ssuis*,_ and the receptor recovered to pretreatment levels on day 2 after treatment. Flow cytometry analysis of B and T cells incubated with fluorescein-labelled recombinant proteins revealed that different rIde_*Ssuis*_ variants bind specifically to B cells, most prominently the cleavage-deficient variant. Our results indicate that in vitro interference of rIde_*Ssuis*_ with the IgM B cell receptor results in long-lasting impaired IgM secretion by B cells after toll-like receptor activation. Further studies are warranted to prove that the modulation of B cell function by Ide_*Ssuis*_ could play a role in vivo.

**Supplementary Information:**

The online version contains supplementary material available at 10.1186/s13567-024-01363-1.

## Introduction

*Streptococcus suis* (*S. suis*) is one of the most important porcine pathogens worldwide [1]. Disease mostly occurs in piglets between 6 and 10 weeks of age and often manifests as polyarthritis, meningitis, endocarditis or septicemia. *S. suis* is not only an invasive pathogen but also a very successful colonizer of mucosal surfaces, especially the upper respiratory tract and tonsils of pigs of all ages [2]. Up to 100% of conventionally farmed pigs are carriers of *S. suis* [3]. Additionally, *S. suis* has important zoonotic potential, with the highest human case rates in Asia [4–6].

Many invasive *S. suis* strains express a variety of different virulence-associated factors, e.g., suilysin or muramidase-released protein (MRP) [7–10]. Modification of lipoteichoic acids and peptidoglycan further contributes to immune evasion [11–13]. However, until now, only the polysaccharide capsule, which determines the classification into 29 different serotypes (cps) [14], has been collectively agreed upon as a critical virulence factor [15, 16]. It provides protection against phagocytosis, masks surface proteins [17–19] and can be modulated within the host [20]. The serotype distribution of *S. suis* differs depending on geographic location [21], but overall, cps 2 is the most important serotype in pigs and humans worldwide [22, 23].

The immunoglobulin M-degrading enzyme of *Streptococcus suis*, designated Ide_*Ssuis*_, is a cysteine protease with homology to the immunoglobulin G-degrading enzyme of *S. pyogenes* (IdeS) [24, 25]. Cleavage of soluble IgM between the constant domains 2 and 3 is an important complement evasion mechanism, as it results in the release of the IgM Fab fragment [26]. The expression of Ide_*Ssuis*_ therefore promotes the survival of *S. suis* in the blood of 7- to 10-week-old piglets, with high levels of IgM binding to the bacterial surface [27]. On the other hand, it has been shown that the expression of Ide_*Ssuis*_ is not crucial for the virulence of an invasive cps 2 strain in vivo [27], so the role of Ide_*Ssuis*_ expression in *S. suis* has not been elucidated.

In our previous work, we showed that Ide_*Ssuis*_ cleaves not only soluble IgM but also the IgM B cell receptor (BCR), leading to impaired BCR signalling after BCR-specific activation immediately after cleavage [28].

The ligand-binding part of the BCR consists of a monomeric membrane-bound immunoglobulin. The IgM BCR is present on B1 cells and porcine B1-like cells, which constitutively secrete IgM as a natural antibody without specific antigen encounters. Naïve B2 cells also express IgM as a BCR before they undergo isotype switching and develop into antigen-specific plasma cells [29–31]. To prevent autoimmune disease, BCR signalling is tightly controlled. Positive coregulators such as CD19 enhance phosphorylation signals. CD22, as a negative coregulatory protein, contains immunoreceptor tyrosine-based inhibitory motifs (ITIM) to recruit protein tyrosine phosphatases (PTP) to downregulate phosphorylation and therefore limit B cell activation [32, 33].

We hypothesize that IgM B cell downregulation by Ide_*Ssuis*_ expression promotes the survival of *S. suis* at local sites such as mucosal surfaces, lymph nodes or tonsils. In this work, we verified IgM BCR cleavage ex vivo in whole regional lymph nodes and investigated the working hypothesis that cleavage of the IgM BCR results in impaired IgM secretion by porcine B cells 3 days after BCR cleavage in an in vitro system.

## Materials and methods

### Bacterial strains and growth conditions

*S. suis* cps 2 strain 10 (wild-type, wt) and the isogenic point-mutant *S. suis* 10Δide_*Ssuis*_∇ide_*Ssuis*__C195S (∇ide_*Ssuis*__C195S), generated in a previous study [27], were grown on Columbia blood agar plates (Thermo Oxoid, Schwerte, Germany, catalogue PB5039A) or in Todd-Hewitt-Broth (THB) (Bacto, BD, Heidelberg, Germany, catalogue 249240) at 37 °C with 5% CO_2_. *Escherichia coli* BL21 or M15 carrying plasmids encoding recombinant proteins were cultured in Luria–Bertani (LB) medium supplemented with 100 mg/mL ampicillin.

### Expression, purification and labelling of recombinant (r) proteins

Recombinant Ide_*Ssuis*__homologue (rIde_*Ssuis*__h), Ide_*Ssuis*__homologue_C195S (rIde_*Ssuis*__h_C195S), Ide_*Ssuis*__C_domain (rIde_*Ssuis*__C) and muramidase-released protein (rMRP) were expressed in *Escherichia coli* BL21 pETIde_*Ssuis*__homologue, BL21 pETIde_*Ssuis*__homologue_C195S, BL21 pETIde_*Ssuis*__C_domain and M15 pQE mrp, respectively. Isopropyl-b-D-thiogalactopyranoside (IPTG) was added during the exponential growth phase, and recombinant proteins were purified through Ni-affinity chromatography as described previously [25, 27]. On the basis of endotoxin measurements performed in a previous study [34], we assumed that the final LPS concentrations were between 0.152 and 0.3 ng/well in the ELISpot experiments.

A portion of the recombinant Ide_*Ssuis*__homologue, Ide_*Ssuis*__homologue_C195S, Ide_*Ssuis*__C_domain and MRP was conjugated with NHS-fluorescein (Thermo Fisher Scientific, Schwerte, Germany, catalogue 46410) following the manufacturer’s instructions. After chromatographic purification, the degree of labelling was calculated from spectrophotometric measurements as specified in the manufacturer’s instructions.

### Purification of immunoglobulin

Purified and cross-adsorbed rabbit anti-porcine IgM F(ab′)_2_ polyclonal antibodies (hereafter referred to as anti-IgM F(ab′)_2_) were generated and fluorescein labelled in a previous study [28].

### Cells

Whole heparinized blood was collected from 7- to 9-week-old commercially farmed piglets (German Landrace × Pietrain).

Preparation of peripheral blood mononuclear cells (PBMC) was performed via density gradient centrifugation over Pancoll, as described previously [28]. The cells were resuspended in Iscove’s modified Dulbecco’s medium (IMDM) supplemented with 40% heat-inactivated FCS and 10% dimethyl sulfoxide (DMSO, Sigma, Darmstadt, Germany, catalogue D2650-100ML), frozen and stored at −80 °C until use.

### BCR cleavage ex vivo in local lymph nodes

Eight- to 9-week-old commercially farmed piglets (German Landrace × Pietrain) were euthanized for the collection of tonsils and other tissues for research purposes in accordance with European and German animal welfare laws. The following local lymph nodes were selected: (i) Ln. cervicalis superficialis dorsalis, (ii) Ln. subiliacus, (iii) Ln. inguinalis superficialis. The skin was incised above the respective lymph node until the lymph node was visible but still had an intact capsule and was embedded in surrounding fatty tissue. The right lymph nodes were injected with rIde_*Ssuis*__h, and the left lymph nodes were injected with rIde_*Ssuis*__h_C195S (both 50 µg/Ln in 140 µL of PBS). Then, the lymph nodes were removed completely and transported to the laboratory in IMDM. Cell isolation was performed as described previously [28]. Briefly, the lymph nodes were minced and transferred into digestion medium (IMDM supplemented with 1% penicillin/streptomycin, 50 µM gentamicin and 111.1 U/mL DNase). After incubation for 15 min at 37 °C, the samples were homogenized using a gentleMACS™ Dissociator according to the manufacturer’s instructions, followed by filtration through sterilized cotton wool and rinsing with PBS. The cells were washed three times with PBS at 400 × *g* and 4 °C for 12 min, stained for BCR cleavage and analyzed by flow cytometry as described above.

### Enzyme-linked immunospot assay (ELISpot)

Porcine PBMC were thawed, counted and adjusted to 1 × 10^6^ cells/mL in IMDM supplemented with 10% heat-inactivated fetal calf serum (FCS), 1% penicillin‒streptomycin (PAN Biotech, Aidenbach, Germany, catalogue P06-07100) and 10 ng/mL recombinant porcine interleukin 2 (poIL-2) (R&D Systems, Minneapolis, US, catalogue 652-P2), which is subsequently referred to as IMDM^++^ + poIL-2. If needed, 0.25 µg/mL R848 (Enzo, Farmingdale, NY, US, catalogue ALX-420-038-M005) was added, following referred to as stimulation medium.

For each sample, duplicates or triplicates of 2 × 10^5^ cells each were transferred into a 96-well plate and treated in one of the following ways: (i) incubation with proteins for 45 min, removal of proteins by washing and incubation for 3 days in stimulation medium, (ii) incubation for 3 days in stimulation medium, incubation with proteins for 45 min and removal of proteins by washing.

The following recombinant proteins were added at a concentration of 4 µg/10^6^ cells: (i) rIde_*Ssuis*__h, (ii) rIde_*Ssuis*__h_C195S, (iii) rIde_*Ssuis*__C, and (iv) rMRP. Following incubation for 45 min at 37 °C with 5% CO_2_, the proteins were removed by washing three times with IMDM.

Ninety-six-well ELISpot plates (MultiScreen^®^ filter plates, Merck Millipore, Darmstadt, Germany, catalogue MSIPS450) were activated by adding 25 µL/well 37% ethanol for 1 min, followed by washing three times with 150 µL/well PBS. The plates were coated overnight with 50 µL/well anti-porcine IgM in PBS (final concentration of 2.5 µg/mL) at 4 °C. Next, the coating antibody was removed, and after three washing steps with PBS, the ELISpot plates were blocked by adding 200 µL/well IMDM supplemented with 10% FCS and incubated for at least one hour at 37 °C.

After 3 days of incubation in stimulation medium, the cells were washed, pooled, counted and adjusted to 1 × 10^5^ cells/mL in IMDM^++^ + poIL-2 (10 ng/mL final conc.). Duplicates or triplicates of 1 × 10^4^ cells/well each were transferred into the ELISpot plate and incubated for 24 h at 37 °C with 5% CO_2_. During this incubation time, every kind of disturbance and vibration was avoided.

The next day, the ELISpot plates were washed two times with distilled water (every subsequent washing step was performed with 200 µL/well) and three times with PBS + 0.01% Tween 20 (Roth, Karlsruhe, Germany, catalogue 9127.1). One hundred microliters/well of goat anti-pig IgM*biotin diluted in PBS + 0.01% Tween 20 + 1% bovine serum albumin (BSA) (Roth, Karlsruhe, Germany, catalogue 8076.3) was added, and the plate was incubated for 1.5 h in the dark at room temperature (RT). After 4 times washing with PBS + 0.01% Tween 20, 100 µL/well streptavidin diluted in PBS + 0.01% Tween 20 + 1% BSA was added, and the plate was incubated for 45 min in the dark at room temperature (RT). After three washing steps with PBS + 0.01% Tween 20 and two washing steps with PBS alone, 100 µL/well alkaline phosphatase substrate (SigmaFast™ BCIP^®^/NBT substrate, Sigma Aldrich, Steinheim, Germany, catalogue B5655-25TAB) (reconstituted according to the manufacturer’s instructions) was added, followed by 5 min of incubation in the dark at RT. Next, the plates were thoroughly rinsed with tap water and dried for one day in the dark.

Spots were detected with an AID vSpot image analyser (AID Diagnostika, Straßberg, Germany). All the antibodies and corresponding dilutions used are specified in Additional file 1.

### ELISA

The supernatants from the ELISpot experiments were collected after the cells were incubated for three days in stimulation medium (as described above), and the amount of secreted soluble IgM was analyzed.

Microtiter plates (U96 MaxiSorp Nunc-Immuno Plate, catalogue 442404, Thermo Scientific, Denmark) were coated with 50 µL/well anti-IgM antibody diluted 1:500 in PBS and incubated overnight at 4 °C. After three times washing, the plates were frozen and stored at −20 °C.

After thawing, the plates were blocked for one hour at room temperature (RT) with 250 µL/well blocking buffer (containing PBS supplemented with 0.5% BSA and 0.1% gelatin), followed by three times washing.

As an IgM standard, whole pig IgM was used at a 100 ng/mL working concentration with a 1:2 serial dilution. Sample dilution was determined individually on the basis of ELISpot data. For dilution, PBS supplemented with 0.5% BSA, 0.1% gelatin and 0.05% Tween 20 (sample diluent) was used. After incubation for 1 h at RT, the plates were washed three times. Next, a biotinylated anti-pig antibody was added (0.1 µg/mL, 50 µL/well) before incubation for 1 h at RT. After three washing steps, 50 µL/well of streptavidin conjugated with horseradish peroxidase was added, and the plates were incubated for 20 min at RT before being washed again five times. TMB peroxidase substrates A and B (SeraCare Life Sciences, Milford, MA, USA, catalogues 5120-0049 and 5120-0038) were mixed in equal parts, 100 µL/well was added, and the plates were incubated in the dark for 8 min. To stop development, 50 mL/well 1 M H_3_PO_4_ was added. Emissions were detected at 450 and 630 nm with a SpectraMax 340PC microplate reader and SoftMax^®^ Pro v5.0 software (both from Molecular Devices, San Jose, CA, USA).

All washing steps were performed with 200 µl/well of PBS supplemented with 0.05% Tween 20 (PBST) with a BioTek^®^ 405 LS microplate washer (BioTek Instruments, Winooski, USA). All the antibodies and corresponding dilutions used are specified in Additional file 2.

### Characterization of PBMC incubated with recombinant proteins

The cells used for flow cytometry analysis were collected from ELISpot experiments. As described above, PBMC were thawed, adjusted and incubated with 4 µg/10^6^ cells of the different recombinant proteins (rIde_*Ssuis*__homologue, rIde_*Ssuis*__homologue_C195S, rIde_*Ssuis*__C_domain or rMRP) or in medium alone. After 45 min of incubation at 37 °C with 5% CO_2_, the recombinant proteins were removed by washing three times with IMDM^++^. Samples were collected immediately after washing (0 h) or two days and three days after resuspension and incubation in stimulation medium.

The cells were stained for viability and intact or cleaved IgM BCR as described previously [28]. Briefly, the cells were transferred to a 96-well plate, washed two times with cold PBS and stained for viability for 20 min at 4 °C. After two washing steps with cold PBS, the cells were fixed for 30 min at 4 °C in 100 µL of 2% paraformaldehyde (PFA). Next, the cells were washed two times with cold FACS buffer (PBS supplemented with 3% heat-inactivated FCS and 0.1% sodium acid), followed by staining for 15 min at 4 °C in 20 µL with anti-IgM Fc and anti-IgM F(ab′)_2_ or the respective isotype controls. The cells were washed two times with cold FACS buffer. After 10 min of incubation in Fc block I (FACS buffer containing 30% heat-inactivated porcine serum), the cells were stained for 15 min at 4 °C in 20 µL with anti-CD3 or the respective isotype control as well as the secondary antibody for anti-IgM Fc. One wash with FACS buffer and two washes with saponin buffer (FACS buffer containing 0.5% saponin) were followed by a 5 min incubation in Fc block II (saponin buffer containing 30% heat-inactivated porcine serum) and intracellular staining for 30 min at 4 °C in 20 µL with anti-CD79α or CD79α fluorescence minus one (FMO) control. Finally, the cells were washed one time with saponin buffer and one time with FACS buffer and were subsequently resuspended in FACS buffer for measurement by flow cytometry (LSRFortessa, BD Biosciences, Heidelberg, Germany).

Flow cytometry analysis was carried out using FlowJo™ 10 software (BD Biosciences, Heidelberg, Germany). Gates were placed on the basis of specific isotype or FMO controls. First, lymphocytes were gated on size and granularity. Next, single cells were gated to exclude doublets. Viable B cells (CD79a^+^CD3^−^) and T cells (CD79a^−^CD3^+^) were distinguished. IgM Fc^+^ B cells were analyzed for the percentage of B cells with an intact (IgM Fc^+^ F(ab′)_2_^+^) or cleaved (IgM Fc^+^ F(ab′)_2_^−^) IgM B cell receptor as well as for the median fluorescence intensity of IgM F(ab′)_2_. The detailed gating strategy is described in [28].

All washing steps were performed for 4 min at 400 × *g* or 500 × *g* after fixation. All antibodies and concentrations used in flow cytometry are specified in Additional file 3.

### Characterization of the effect of R848 on B cell activation

To further characterize the effect of R848 as component of the stimulation medium on B cells, porcine PBMC were thawed, counted and adjusted to 1 × 10^6^ cells/mL in IMDM^++^ medium supplemented either with only porcine IL-2 (10 ng/mL) or with both porcine IL-2 and R848 (0.25 µg/mL), followed by incubation at 37 °C and 5% CO_2_ for up to 3 days. Samples were collected before the addition of IL-2 or IL-2 and R848 (untreated control), as well as after one, two, and three days of incubation. The cells were stained for viability and fixed as described above. Staining mixture 1 contained anti-CD25 or the respective isotype control. All other staining and washing steps were performed as described above. The percentage and MFI of CD25 + B cells were analysed by flow cytometry (LSRFortessa and FlowJo™ 10 software, both BD Biosciences, Heidelberg, Germany). All antibodies and concentrations used in flow cytometry are specified in Additional file 3.

### Incubation of PBMC with FITC-labelled recombinant proteins

PBMC were thawed, counted and adjusted to 1 × 10^6^ cells/mL in IMDM^++^ medium. The cells were either treated with 4 µg/10^6^ FITC-labelled rIde_*Ssuis*__homologue, rIde_*Ssuis*__homologue_C195S, rIde_*Ssuis*__C_domain or rMRP, respectively, or were kept in cell culture medium as a control. After 45 min, the FITC-labelled recombinant proteins were removed by 2 washing steps with PBS.

One part of the cells was directly fixed by adding 2% paraformaldehyde (PFA) for 30 min at 4 °C, followed by washing with cold FACS buffer. After a total of 4 washing steps, the cells were directly analysed by flow cytometry to determine the percentage of FITC-positive single cells within the lymphocyte population. The median fluorescent intensity (MFI) in the FITC channel of all single cells was classified as autofluorescence. The MFI of the FITC^+^ single cells was taken as the specific fluorescence due to incubation with the FITC-labelled recombinant proteins.

The other part of the cells was washed a total of 14 times to discriminate between weak and strong (potentially specific) binding, stained for viable B cells as described above and analysed for the percentage of FITC^+^ IgM Fc^+^ B cells. The MFI of the FITC signal of all the IgM Fc^+^ B cells was classified as autofluorescence. The MFI of FITC^+^ IgM Fc^+^ B cells was taken as the specific fluorescence.

All washing steps were performed for 4 min at 400 × *g* or 500 × *g* after fixation. All antibodies and concentrations used are specified in Additional file 3.

### Statistical analysis

All the data represent at least two to three independent experiments. Statistical analysis was performed with GraphPad Prism 9 (Dotmatics, Boston, Massachusetts, USA; [35]). The data distribution was tested with both the Shapiro‒Wilk test and the Kolmogorov‒Smirnov test. If a normal distribution was applied, multiple comparisons were performed via mixed analysis of variance (ANOVA) with subsequent Tukey’s multiple comparisons test. If the data were not normally distributed, the Friedmann test with Dunn’s multiple comparisons test was used. The figures show the means and standard deviations or medians. Probabilities less than 0.05 were considered significant (*p* < 0.05 *, *p* < 0.01 **, *p* < 0.001 ***, *p* < 0.0001 ****). Flow cytometric data were analysed with FlowJo™ 10 software (BD Biosciences, Heidelberg, Germany, [36]).

## Results

### IgM BCR is cleaved by rIde_*Ssuis*__h in local lymph nodes ex vivo

**Figure 1:**
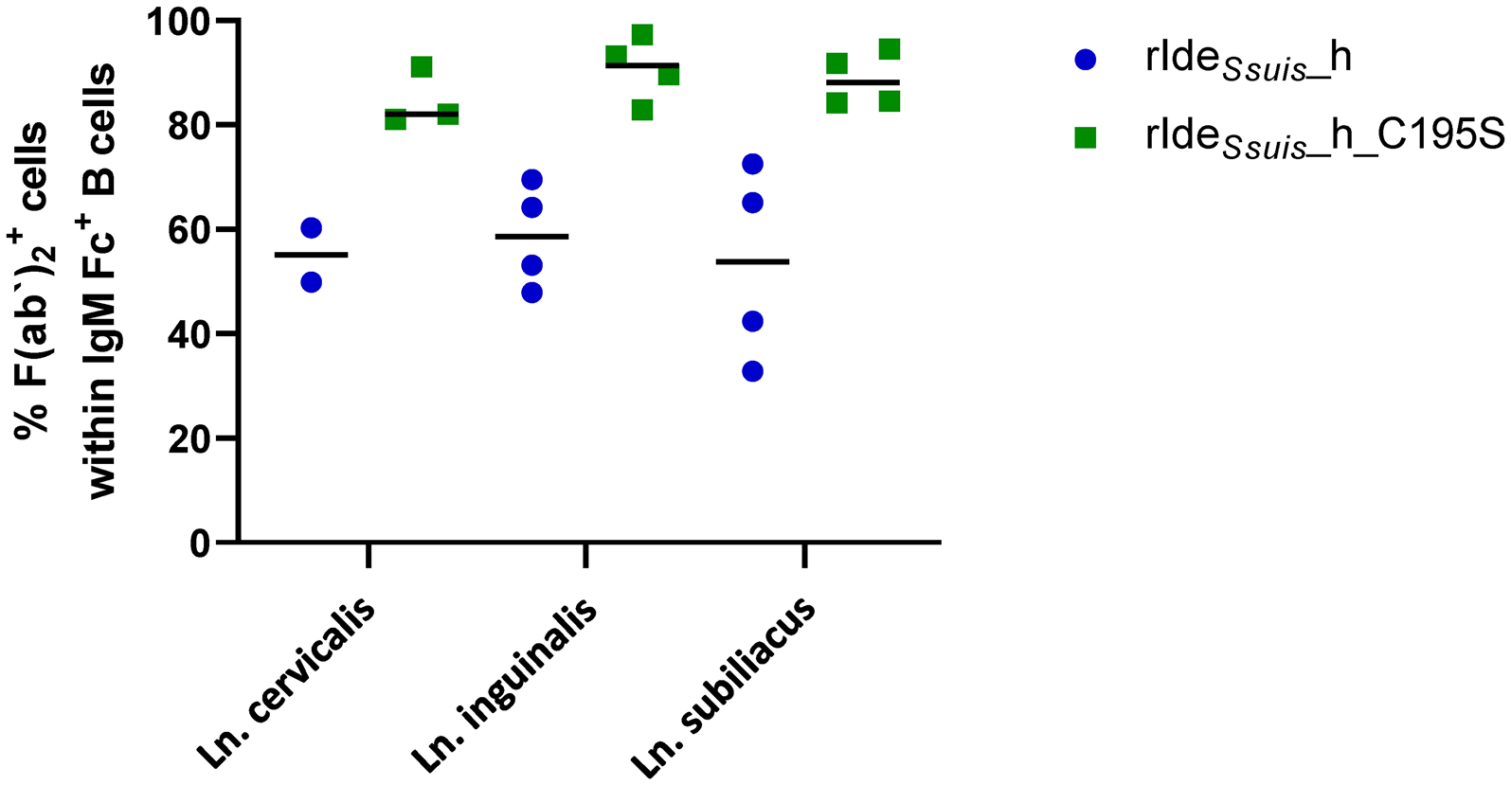
**The IgM BCR is cleaved by rIde**_***Ssuis***_**_h in local lymph nodes ex vivo**. Regional lymph nodes (Ln. cervicalis superficialis dorsalis, Ln. subiliacus, Ln. inguinalis superficialis) were injected with rIde_*Ssuis*__h or rIde_*Ssuis*__h_C195S ex vivo, and the percentage of IgM Fc^+^ F(abˈ)_2_^+^ B cells among the isolated cells was investigated via flow cytometry.

On the basis of the results of our previous study showing that Ide_*Ssuis*_ cleaves not only soluble IgM but also the IgM B cell receptor [28], we wanted to verify these in vitro findings in a model more closely related to the situation in vivo. Therefore, we injected rIde_*Ssuis*__h or rIde_*Ssuis*__h_C195S into different regional lymph nodes (Ln. cervicalis superficialis dorsalis, Ln. subiliacus, Ln. inguinalis superficialis) and investigated IgM BCR cleavage ex vivo. Injecting rIde_*Ssuis*__h reduced the proportion of B cells with intact surface IgM BCR to less than 60% in all investigated lymph nodes (Figure 1). In comparison, between 80% and 100% of B cells from lymph nodes injected with rIde_*Ssuis*__h_C195S exhibited intact surface IgM BCR, which was comparable to the results of in vitro cleavage experiments [28]. These data, for the first time, demonstrate BCR cleavage ex vivo in whole lymph nodes by a streptococcal immunoglobulin protease.

### Incubation with rIde_*Ssuis*__h and rIde_*Ssuis*__h_C195S before stimulation reduces the number of IgM-secreting cells

In our previous work, we reported that IgM BCR cleavage by rIde_*Ssuis*__h results in immediate interference with B-cell signalling [28]. Now, we investigated the impact of IgM BCR cleavage after a longer time period of up to three days, with the readout of IgM secretion after B-cell activation by the Toll-like receptor (TLR) 7/8 ligand R848 (also known as resiquimod) [31].

**Figure 2:**
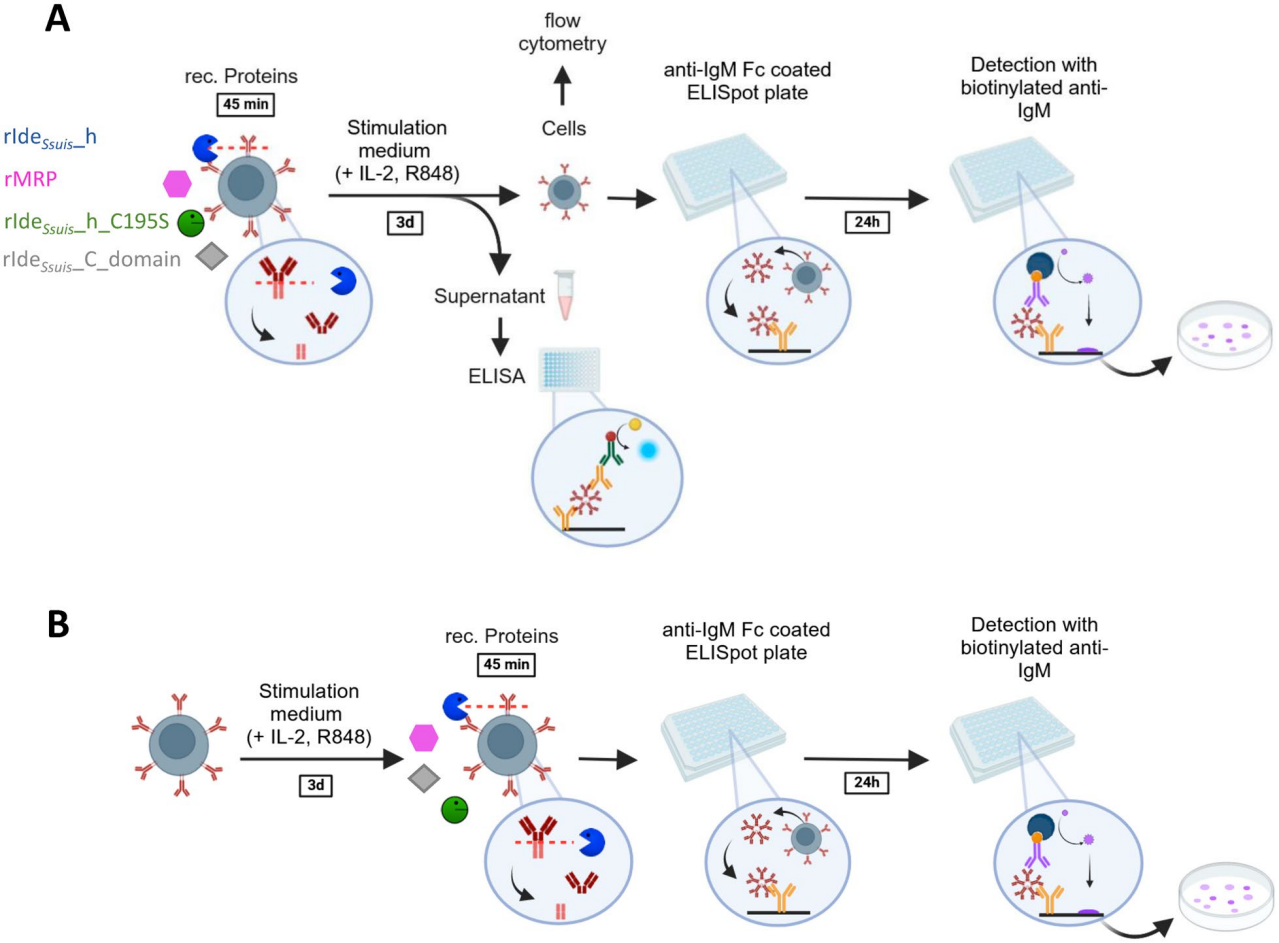
**ELISpot procedure to analyze the number of IgM-secreting cells**. Porcine PBMC were incubated in cell culture medium alone or with (i) rIde_*Ssuis*__homologue (rIde_*Ssuis*__h), (ii) rIde_*Ssuis*__homologue_C195S (rIde_*Ssuis*__h_C195S), (iii) rIde_*Ssuis*__C_domain, or (iv) rMRP for 45 min before protein removal by washing. Treatment with these recombinant proteins was performed before (**A**) or after (**B**) 3 d of incubation in stimulation medium (supplemented with IL-2 and R848). After protein treatment and subsequent stimulation, the culture supernatant was collected for IgM ELISA, and the cells that were not used for ELISpot analysis were analysed via flow cytometry. The cells were washed and readjusted before being seeded in the ELISpot plate at 1 × 10^4^ cells/well. The wells were coated with an anti-IgM antibody to capture secreted IgM. After 24 h, the cells were removed, and the captured IgM was detected with a second, biotinylated anti-IgM antibody. Streptavidin transforms the added substrate to a purple color, which forms visible spots exactly at the place where an IgM-secreting cell has been. This figure was created with Biorender.

Porcine PBMC were treated with different recombinant proteins (cleavage-active rIde_*Ssuis*__h, cleavage-deficient isogenic point-mutated rIde_*Ssuis*__h_C195S, cleavage-deficient rIde_*Ssuis*__C, and rMRP) before (Figure 2A) or after (Figure 2B) 3 days of stimulation, and the number of IgM-secreting cells was determined via ELISpot analysis.

**Figure 3:**
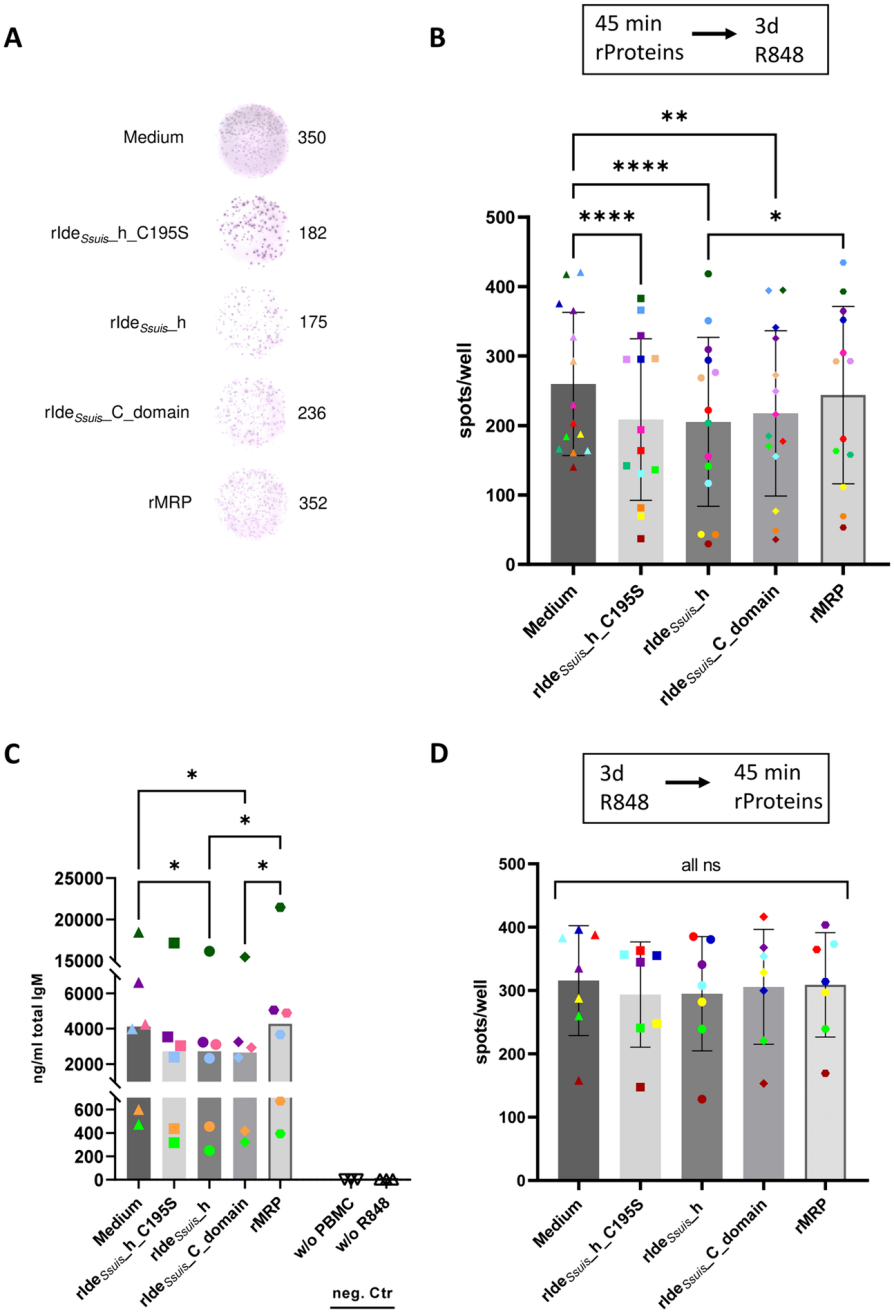
**ELISpot and ELISA analysis of porcine cells after treatment with different recombinant proteins**. **A**,** B** Incubation with rIde_*Ssuis*__homologue and rIde_*Ssuis*__homologue_C195S before stimulation for 3 days reduced the number of IgM-secreting cells. Porcine PBMC were incubated in cell culture medium alone or with (i) rIde_*Ssuis*__homologue (rIde_*Ssuis*__h), (ii) rIde_*Ssuis*__homologue_C195S (rIde_*Ssuis*__h_C195S), (iii) rIde_*Ssuis*__C_domain or (iv) rMRP (all 4 µg/10^6^ cells, 45 min) before protein removal by washing and subsequent incubation in stimulation medium (supplemented with IL-2 and R848) for 3 days. **A** Exemplary ELISpot wells. **B** ELISpot analysis of porcine PBMC (*n* = 14). Statistical analysis was conducted with ordinary one-way ANOVA with Tukey’s multiple comparisons test (*p* < 0.001 ***, *p* < 0.01 **, *p* < 0.05 *, *p* > 0.05 ns). Only comparisons with *p* < 0.05 are shown. The bars and error bars represent the means and standard deviations, respectively. **C** Incubation with rIde_*Ssuis*__homologue and rIde_*Ssuis*__homologue_C195S before stimulation for 3 days also reduced the level of secreted IgM. IgM ELISA of ELISpot culture supernatants collected after 3 days of incubation in stimulation medium. Stimulation medium without PBMC or supernatant from unstimulated cells served as a negative control. Statistical analysis was conducted with the Friedmann test with Dunn’s multiple comparisons test (*p* < 0.05 *, *p* > 0.05 ns). Only comparisons with *p* < 0.05 are shown. The bars represent the medians. **D** Incubation with recombinant proteins after 3 days of stimulation did not impair the number of IgM-secreting cells. ELISpot analysis of porcine PBMC (*n* = 7). The cells were incubated in stimulation medium for 3 days before being washed and incubated in cell culture medium alone or with different recombinant proteins (as described above). Statistical analysis was conducted with ordinary one-way ANOVA and Tukey’s multiple comparisons test (*p* > 0.05 ns). Only comparisons with *p* < 0.05 are shown. The bars and error bars represent the means and standard deviations, respectively. Note: Symbols represent different treatments, and colours represent individual pigs.

Figure 3A shows ELISpot wells exemplary of an experiment in which cell treatment was performed prior to a 3-day stimulation period. When the cells were counted after treatment for 45 min with rIde_*Ssuis*__h prior to three days of incubation in stimulation medium, the number of IgM-secreting cells significantly decreased to a mean of 205.2 spots/well (standard deviation (SD) = 121.7) compared with the mean of 259.8 spots/well (SD = 103) in the corresponding medium control (Figure 3B). The number of rIde_*Ssuis*__h-treated cells was also significantly lower than the mean of 243.8 spots/well (SD = 127.6) of rMRP-treated cells.

Additionally, treatment of cells with rIde_*Ssuis*__C, which was previously found incapable of cleaving IgM [25], resulted in a reduced number of IgM-secreting cells to a mean of 217.4 spots/well (SD = 118.9) compared with the medium control. Interestingly, treatment with cleavage-deficient point-mutated rIde_*Ssuis*__h_C195S [27] also decreased the number of IgM-secreting cells to a mean of 208.6 spots/well (SD = 116.2), comparable to treatment with rIde_*Ssuis*__h.

In contrast, when cells were treated for 45 min with different recombinant proteins (rIde_*Ssuis*__h, rIde_*Ssuis*__h_C195S, rIde_*Ssuis*__C, or rMRP) after 3 days of stimulation with IL-2 and R848, there was no difference in the number of IgM-secreting cells compared with that in cells treated with only cell culture medium (Figure 3D).

We conducted a further independent ELISpot experiment with PBMCs from different piglets to verify these results and to measure secreted IgM. Total IgM ELISA analysis of ELISpot culture supernatants revealed significantly reduced levels of secreted IgM after cells were treated with rIde_*Ssuis*__h or rIde_*Ssuis*__C (Figure 3C). Without stimulation, cells do not spontaneously secrete IgM. Notably, pigs with the lowest numbers of IgM-secreting cells also presented the lowest levels of secreted IgM. Taken together, these data demonstrate that, when incubated prior to a 3-day stimulation period, different rIde_*Ssuis*_ variants, especially rIde_*Ssuis*__h and rIde_*Ssuis*__h_C195S, reduce not only the number of IgM-secreting porcine B cells but also the secretion of total IgM.

### The expression of the IgM BCR on PBMC reaches levels comparable to those of cells treated with medium or other recombinant proteins two days after treatment with rIde_*Ssuis*__h

**Figure 4:**
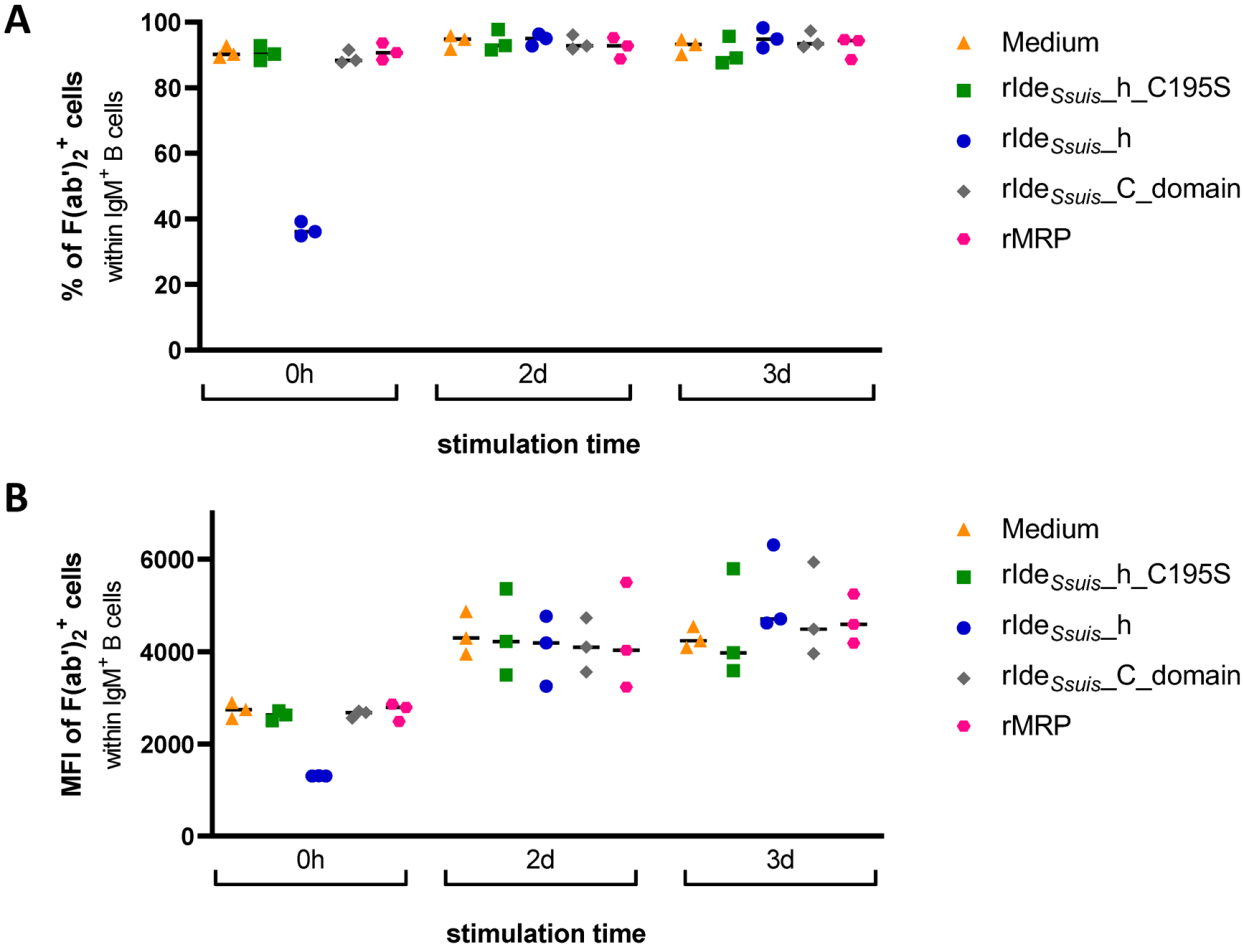
**Expression of the IgM BCR on PBMC reaches levels comparable to those of cells treated with medium or other recombinant proteins two days after treatment with the rIde**_***Ssuis***_**_homologue**. Porcine PBMC were incubated in cell culture medium alone or with (i) rIde_*Ssuis*__homologue (rIde_*Ssuis*__h), (ii) rIde_*Ssuis*__homologue_C195S (rIde_*Ssuis*__h_C195S), (iii) rIde_*Ssuis*__C_domain or (iv) rMRP (all 4 µg/10^6^ cells, 45 min) before protein removal by washing. Samples were taken immediately after washing (0 h) or after 2 days or 3 days of incubation in stimulation medium (supplemented with IL-2 and R848). The cells were stained and analysed for the percentage of IgM Fc^+^ F(abˈ)_2_^+^ B cells (**A**) and the respective median fluorescence intensity (MFI, **B**).

To confirm BCR cleavage by rIde_*Ssuis*__h and exclude possible interference of other cleavage-deficient recombinant proteins (rIde_*Ssuis*__h_C195S, rIde_*Ssuis*__C, and rMRP) with BCR expression, we performed flow cytometry analysis. Cells from representative ELISpot experiments were analysed for surface IgM BCR expression at different time points. Figure 4 shows the respective flow cytometry data. Immediately after treatment with rIde_*Ssuis*__h (t = 0 h), the percentage of IgM F(abˈ)_2_^+^ Fc^+^ B cells decreased from 90% to 36%, demonstrating the expected IgM BCR cleavage by cleavage-active rIde_*Ssuis*__h but not by the other cleavage-deficient rIde_*Ssuis*_ constructs or by the unrelated rMRP *S. suis* control protein (Figure 4A). The matching median fluorescence intensity (MFI) values of IgM F(abˈ)_2_^+^ Fc^+^ B cells after treatment with rIde_*Ssuis*__h were also reduced (from 2744 to 635), indicating reduced IgM BCR surface expression per cell (Figure 4B). After two days, the percentage of IgM F(abˈ)_2_^+^ Fc^+^ B cells as well as the corresponding MFI values in PBMC previously treated with rIde_*Ssuis*__h returned to levels comparable to those in cells treated with only medium or the other recombinant proteins and remained at that level at the 3-day time point, demonstrating that the IgM BCR recovered on the cell surface. Overall, the IgM BCR surface expression in cells treated with medium, rIde_*Ssuis*__h_C195S, rIde_*Ssuis*__C or rMRP did not differ. Additionally, we observed a general increase in MFI values over time. Hence, we further characterized the effects of the components of the stimulation medium, i.e., recombinant porcine IL-2 and R848, on B cells and chose CD25, which is a part of the IL-2 receptor, as a marker for B cell activation [37]. Flow cytometry analysis revealed a time-dependent increase in the percentage of CD25^+^ B cells from a mean of 2.99 ± 1.3% (untreated control) to 30.1 ± 10.8% on day 3 when porcine IL-2 was supplemented in the cell culture medium and further increased to 44.9 ± 8.4% when R848 was present in the medium. Similarly, adding IL-2 led to an increase in the MFI of CD25^+^ B cells to 1597 ± 128.9 on day 3 compared with that of medium-treated cells (MFI of 997 ± 32.6), and additional supplementation with R848 further increased the MFI to 2117 ± 201.4 (Additional file 4). Thus, we confirmed R848 as a potent inducer of CD25 upregulation and concluded that the increase in the MFI of IgM F(abˈ)_2_^+^ Fc^+^ B cells over time was due to general B cell activation.

**Figure 5:**
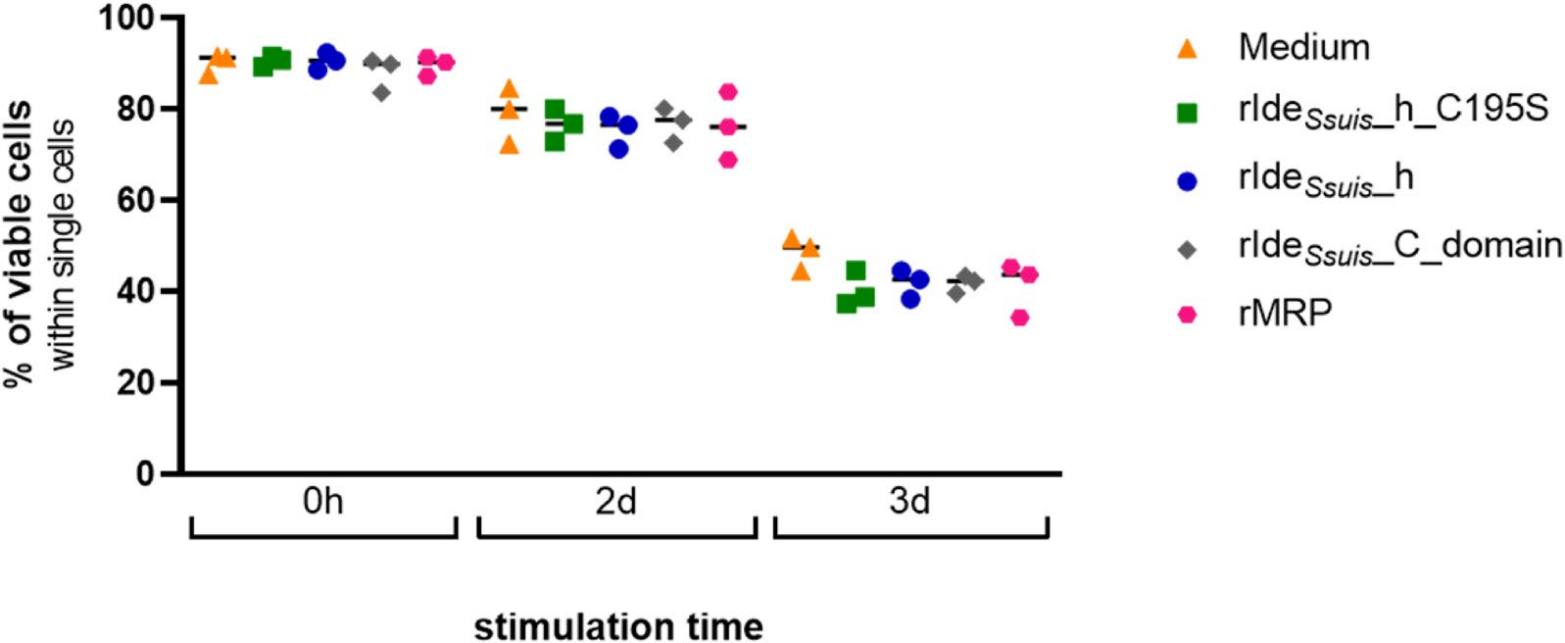
**Incubation with recombinant proteins does not impair cell viability**. Porcine PBMC (*n* = 3, representative data points) were incubated in cell culture medium alone or with (i) rIde_*Ssuis*__homologue (rIde_*Ssuis*__h), (ii) rIde_*Ssuis*__homologue_C195S (rIde_*Ssuis*__h_C195S), (iii) rIde_*Ssuis*__C-domain or iiii) rMRP (all 4 µg/10^6^ cells, 45 min) before protein removal by washing. Samples were taken immediately after washing (0 h) or after 2 days or 3 days of incubation in stimulation medium (supplemented with IL-2 and R848). The cells were stained with live‒dead stain and analysed for viable cells within single lymphocytes via flow cytometry.

Finally, we determined the viability of the cells directly after 45 min of treatment with the different recombinant proteins or after incubation in stimulation medium for up to 3 days. Flow cytometry analysis revealed a general, time-dependent decrease in the percentage of viable cells from 90% to approximately 45% on day 3, but no differences were detected between cells treated with only medium or the different recombinant proteins (Figure 5). Viability after 3 days of incubation was also verified for every ELISpot assay with trypan blue, and the results were similar to those of the flow cytometric viability analysis (data not shown). This finding demonstrated that the observed reduction in IgM B-cell activation caused by the rIde_*Ssuis*__variants was not due to the loss of cells caused by cell death.

### The cleavage-deficient point mutant rIde_*Ssuis*__h_C195S binds more strongly to IgM^+^ B cells than rIde_*Ssuis*__h

ELISpot analysis revealed a significant decrease in the number of IgM-secreting cells after treatment not only with rIde_*Ssuis*__h but also with other cleavage-deficient variants of rIde_*Ssuis*_ (Figure 3B). This was not due to decreased cell viability (Figure 5) or interference with surface IgM BCR expression caused by treatment with rIde_*Ssuis*__h_C195S (Figure 4).

Therefore, we hypothesized that the different rIde_*Ssuis*_ variants specifically target B cells, leading most likely to interfere with BCR signalling. Accordingly, we measured the binding of fluorescein isothiocyanate (FITC)-labelled recombinant proteins to B and T cells.

**Figure 6:**
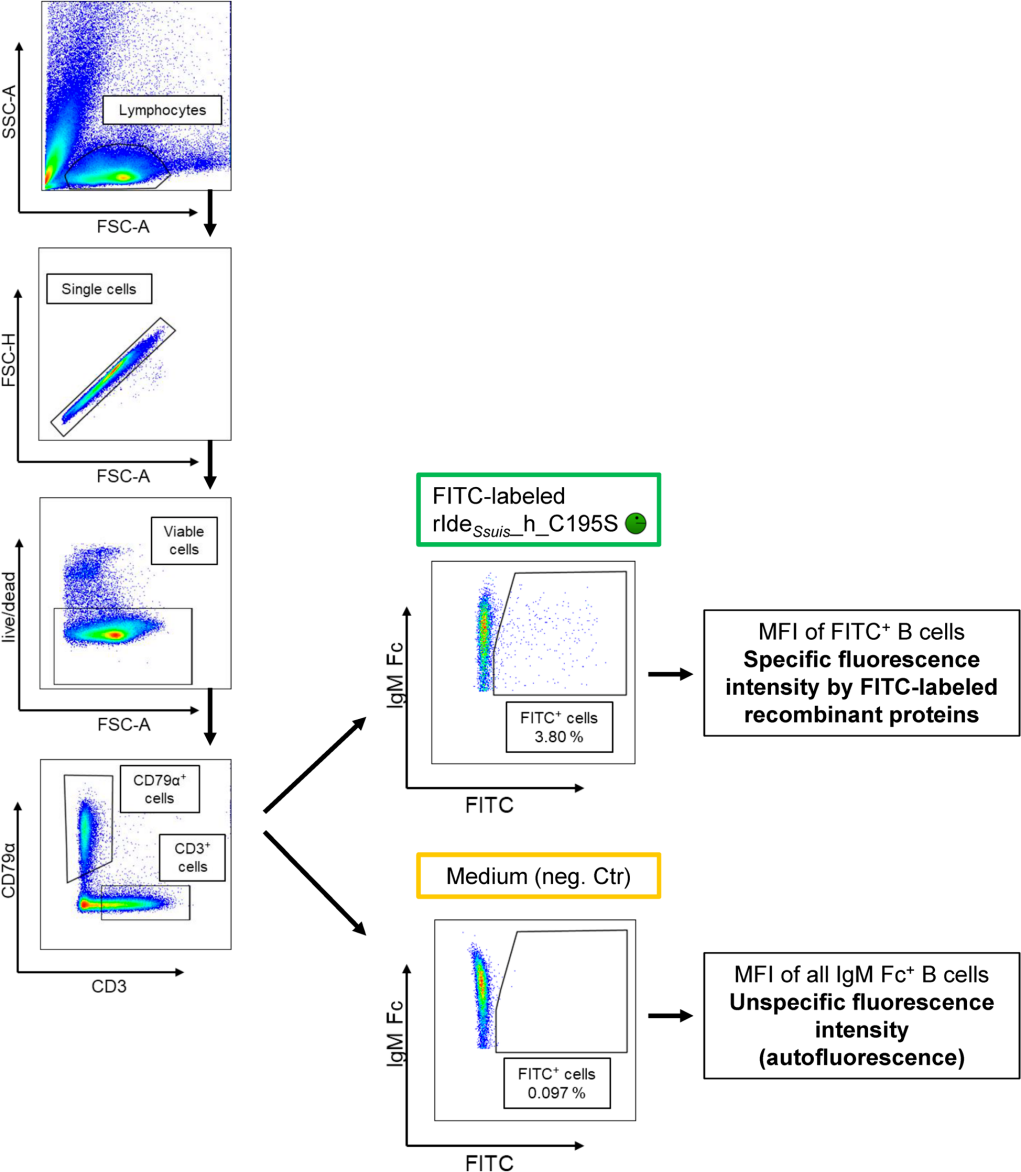
**Incubation with FITC-labelled recombinant proteins: Gating strategy**. Representative pseudocolor plots are shown. Porcine PBMC were incubated in cell culture medium alone (negative control) or with FITC-labelled recombinant proteins (4 µg/10^6^ cells, 45 min). Viable cells were gated for the B-cell marker CD79α and surface IgM. Finally, the percentage of FITC^+^ IgM + B cells was analysed. The median fluorescence intensity (MFI) of IgM^+^ B cells incubated in medium alone was considered autofluorescence. The MFI of the FITC^+^ IgM^+^ B cells was considered to be specific because they were incubated with the FITC-labelled proteins.

After 45 min of incubation, the percentage of FITC^+^ cells and the respective MFI of the cells were analyzed by flow cytometry. The cells were either washed 4 times (for analysis directly after fixation) or 14 times (for staining for the B cell marker CD79a and surface IgM) to assess weak and stronger (potentially specific) binding. Figure 6 shows the gating strategy for the latter. The MFI of all IgM Fc^+^ B cells incubated in medium alone accounted for unspecific autofluorescence. The MFI of FITC^+^ IgM Fc^+^ B cells after incubation with FITC-labelled proteins was considered to be specific because the FITC-labelled protein bound to the IgM BCR.

**Figure 7:**
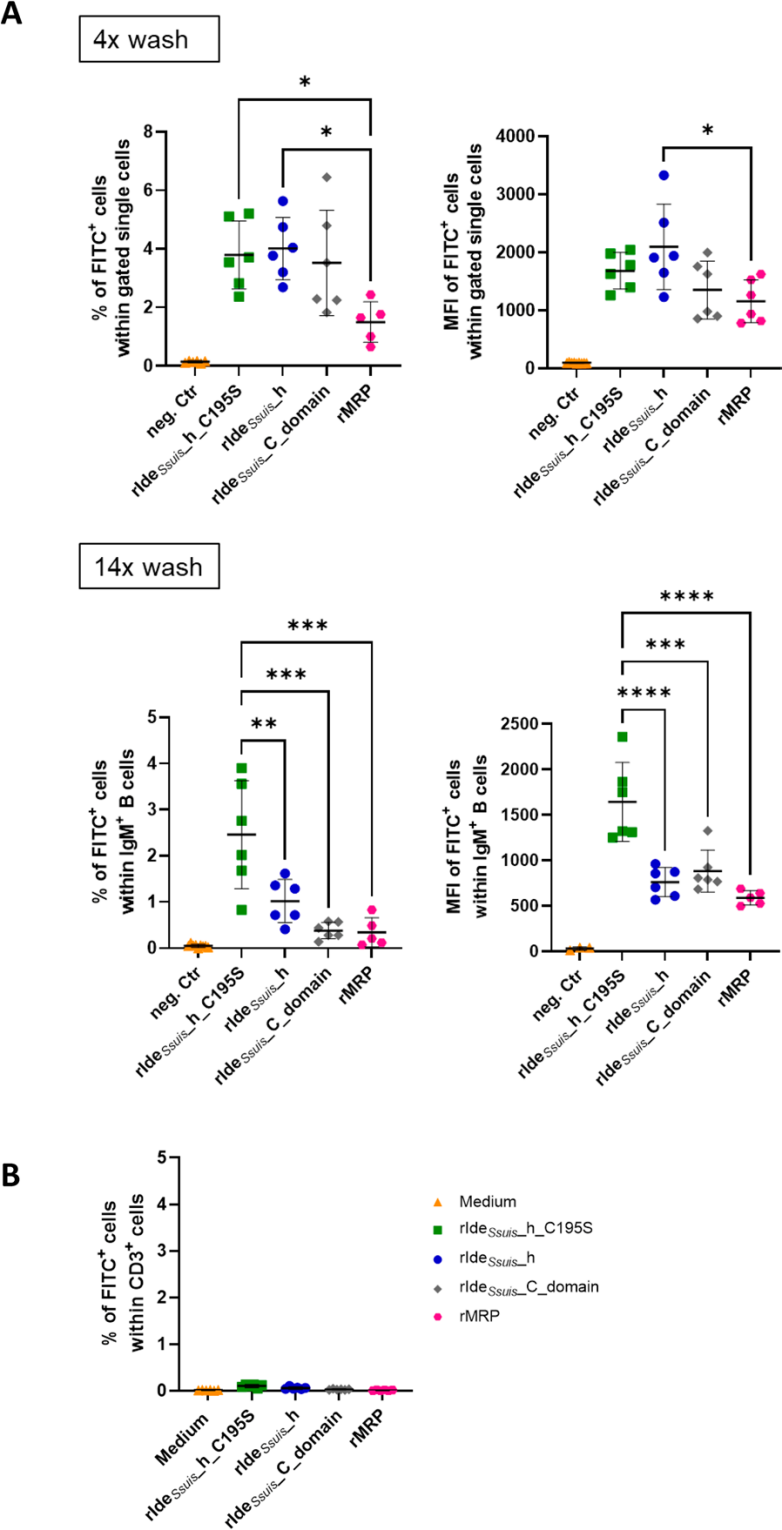
**Cleavage-deficient point-mutant rIde**_***Ssuis***_**_homologue_C195S binds more strongly to IgM**^**+**^ **B cells than does rIde**_***Ssuis***_**_homologue.** **A** Flow cytometry analysis of FITC^+^ cells. Porcine PBMC (*n* = 6) were incubated with FITC-labelled proteins: (i) rIde_*Ssuis*__homologue (rIde_*Ssuis*__h), (ii) rIde_*Ssuis*__homologue_C195S (rIde_*Ssuis*__h_C195S), (iii) rIde_*Ssuis*__C_domain or (iv) rMRP (all 4 µg/10^6^ cells, 45 min). Upper row: Cells were analyzed directly after fixation, with a total of 4 washing steps before analysis. Lower row: Cells were stained for IgM^+^ B cells for a total of 14 washing steps before analysis. The percentage of FITC^+^ cells (left hand side) and the respective median fluorescence intensity (MFI, right hand side) are shown. Statistical analysis was conducted with ordinary one-way ANOVA with Tukey’s multiple comparisons test (*p* < 0.0001 ****, *p* < 0.001 ***, *p* < 0.01 **, *p* < 0.05 *, *p* > 0.05 ns). Only comparisons with *p* < 0.05 are shown. The bars and error bars represent the means and standard deviations, respectively. **B** FITC-labelled recombinant proteins do not bind to CD3^+^ T cells. Porcine PBMC (*n* = 6) were incubated in cell culture medium alone or with the indicated recombinant proteins as described above. The CD3 + T cells were stained and analysed by flow cytometry (for a total of 14 washing steps before analysis).

After 4 washing steps, flow cytometry analysis revealed no significant difference in the percentage of FITC^+^ cells treated with FITC-labelled rIde_*Ssuis*__h, rIde_*Ssuis*__h_C195S or rIde_*Ssuis*__C and therefore in the binding of these recombinant proteins to viable single cells (Figure 7A, upper row, left panel). In comparison, there were significantly fewer FITC^+^ cells after incubation with FITC-labelled rMRP than after incubation with rIde_*Ssuis*__h or rIde_*Ssuis*__h_C195S. Additionally, the matching MFI of FITC^+^ cells after incubation with FITC-labelled rMRP was significantly lower than that after incubation with FITC-labelled rIde_*Ssuis*__h, whereas there was no significant difference between cells treated with the other FITC-labelled recombinant proteins (Figure 7A, upper row, right panel).

After 14 washing steps, there were significantly more FITC^+^ IgM^+^ B cells when incubated with rIde_*Ssuis*__homologue_C195S than with the other recombinant FITC-labelled proteins (Figure 7A, lower row, left panel). The MFI of FITC^+^ IgM^+^ B cells was also significantly greater when the cells were treated with the point-mutated protein (Figure 7A, lower row, right panel). Moreover, there was no significant difference in the percentage or MFI of FITC^+^ IgM^+^ B cells treated with FITC-labelled rIde_*Ssuis*__h, rIde_*Ssuis*__C or rMRP.

To ensure binding specificity, PBMC treated with FITC-labelled recombinant proteins were also analyzed for the percentage of FITC^+^ CD3^+^ T cells. Figure 7B shows almost no binding of the recombinant proteins to T cells.

These data demonstrate that the cleavage-deficient point mutant rIde_*Ssuis*__h_C195S binds more strongly to IgM^+^ B cells than rIde_*Ssuis*__h does, whereas rIde_*Ssuis*__C and rMRP are efficiently removed from IgM^+^ B cells after multiple washing steps. In summary, these data indicate that the conserved N-terminal domain of Ide_*Ssuis*_ targets porcine B cells. Furthermore, different truncated recombinant constructs of Ide_*Ssuis*_ also reduce the number of IgM-secreting cells. IgM cleavage is not necessary for this phenotype.

## Discussion

Ide_*Ssuis*_ specifically cleaves soluble porcine IgM as well as the IgM BCR [25, 28]. In this study, we showed that in porcine PBMC,  interaction of Ide_*Ssuis*_ with B cells results not only in immediate but also in longer-lasting interference with IgM B cell activation. We demonstrated the functional consequences for the development of TLR7/8-induced IgM-secreting cells and the corresponding levels of secreted soluble IgM.

Järnum et al. reported similar findings for IgG BCR cleavage by the IgG-degrading enzyme of *Streptococcus pyogenes* (IdeS) [38]. Cleavage of the IgG BCR prior to 3 days of stimulation with R848 led to a significant decrease in the number of IgG-secreting cells, whereas the addition of IdeS after 3 days of stimulation had no effect. We reported analogous findings for IgM BCR cleavage by Ide_*Ssuis*__h. Interestingly, point-mutated rIde_*Ssuis*__h_C195S, which has been proven to cleave neither soluble IgM nor the IgM BCR [27, 28], also reduced the number of IgM-secreting B cells. Thorough washing after incubation of the FITC-labelled rIde_*Ssuis*_ variants with porcine B cells revealed very prominent binding of the point-mutated protein. This finding is in agreement with a study by Wenig et al., where the authors used the point mutant IdeS_C94S for crystallization because it still binds IgG while being more stable than the proteolytically active form [39]. We consider this binding sufficient to interfere with B cell signalling and subsequent IgM secretion.

Compared with the IgG proteases IdeS, IdeE and IdeZ of *S. pyogenes*, *S. equi* subsp. *equi* and *S. equi* subsp. *zooepidemicus*, respectively, which have molecular weights between 35 and 45 kDa, the IgM protease Ide_*Ssuis*_ with 124 kDa in size is a much larger protein [24, 25, 40]. Amino acids 35 to 432 of Ide_*Ssuis*_, which are present in rIde_*Ssuis*__h, are sufficient for IgM cleavage [25]. The function of the C-terminal region, which includes more than 700 amino acids, is not known [25]. The results of our previous study [28] and this study show that the C-terminus is not crucial for the interaction of the IgM cleavage domain with the IgM BCR, as the construct rIde_*Ssuis*__h is sufficient to cleave the IgM BCR and to significantly reduce the number of IgM-secreting cells in vitro.

ELISpot analysis was originally established to analyse specific antibody-secreting B cells [41]. In porcine immunology and vaccine development, ELISpot has mostly been used to investigate the immune response after viral infection, with in vitro restimulation using viral antigens [42–46], and to investigate cellular immune responses to evaluate immunization [47, 48]. Other studies reported frequencies of IgG-secreting cells after 3 days of stimulation with R848 and IL-2 in the range of 392.2 ± 120.5 and approx. 300 ± 140 after an initial cell number of 1 × 10^5^ or 2.5 × 10^4^ PBMC/well, respectively, which is comparable to the results of our investigation of IgM-secreting cells [49, 50].

The limitation of the ELISpot assay is that there is no possibility of discriminating between different IgM-secreting B-cell subpopulations, especially B1 cells and plasma cells [51]. CD25 is expressed as the α-chain of the IL-2 receptor on activated T and B cells [37]. In mice, CD25^+^ B cells spontaneously secrete immunoglobulins [52]. In humans, CD25 expression on B cells has been identified as a marker for memory or regulatory B cells [53, 54]. For porcine B cells, CD25 is also described as an activation marker [31]. Here, R848 stimulation led to the upregulation of CD25 expression by B cells in our ELISpot experiments; therefore, it is likely that most IgM is produced by activated mature B cells.

As porcine B cells do not spontaneously secrete IgM, we chose R848 to induce antibody secretion for ELISpot analysis [31]. R848 is a low-molecular-weight imidazoquinolone that induces cytokine production in dendritic cells, monocytes and macrophages and was therefore first described as a potent antiviral and antitumour agent in animals [55–57]. Later, R848 was shown to function as a TLR7/8 agonist [58]. Via the c-Jun kinase and p38 pathways, similar to CD40 ligand, it stimulates antibody secretion and the upregulation of surface molecules as well as induces proliferation in murine and human B cells [59, 60]. There are several well-established protocols for in vitro polyclonal B-cell activation using R848 to analyse antibody-secreting cells in ELISpot analysis for human [61–63] or porcine cells [42, 49, 50]. Braun et al. characterized the response of porcine B cells to different TLR ligands in detail [31]. They reported that TLR7 is expressed on all B cell subsets and confirmed that R848 is an efficient inducer of B cell activation in porcine B cells. Although R848 B cell activation is a commonly used model, it does not completely reflect the circumstances in vivo when a B cell encounters a specific antigen via its BCR (likely with simultaneous TLR-mediated costimulation). Nevertheless, the results obtained with R848 stimulation provide valuable insights into possible interactions between the host immune system and the pathogen.

Our ELISpot data revealed a reduction in IgM-secreting cells 3 days after treatment with rIde_*Ssuis*__h or rIde_*Ssuis*__h_C195S. Moreover, surface expression of the IgM BCR fully recovered on day 2 after receptor cleavage. This finding suggests that not only cleavage but also strong binding of the point-mutated rIde_*Ssuis*__h_C195S results in comparable impairment of intracellular signalling, which still persists even when surface IgM BCR expression is restored. Different underlying mechanisms are conceivable. One could speculate that cleavage of the IgM BCR by rIde_*Ssuis*_ at a site distal from the disulfide bond between the IgM heavy chains (generating disulfide-bound F(ab)ˈ_2_ fragments) leads to possible dissociation of the remaining truncated heavy chain domains 3 and 4. As the signal-transducing Igα/β dimer needs the BCR to assemble [64, 65], this BCR dissociation may ultimately lead to a loss of Igα/β function. Heterodimeric Igα/β is part of an intact BCR composition and has been shown to be essential for signal transduction by cytoplasmic ITAMs (immunoreceptor tyrosine-based activation motifs) [66, 67]. It remains to be shown how the observed strong binding of rIde_*Ssuis*__h_C195S to IgM B cells inhibits IgM BCR signalling.

The human pathogen *S. pyogenes* expresses the IgG protease IdeS and is a common commensal of the human pharynx, similar to *S. suis* in pigs. Karlsson et al. reported much higher levels of IgG cleavage products in tonsillitis swabs than in plasma samples from sepsis patients, indicating efficient IgG cleavage in IgG-low microenvironments and highlighting the strong adaptation of *S. pyogenes* to its ecological niche [68]. Similarly, the tonsil is an important niche for *S. suis*, as well as a possible site of infection [69]. Additionally, *S. suis* can be detected frequently in various lymph nodes of naturally infected pigs, indicating that the lymph node is an important side of the host‒pathogen interaction and an important immune checkpoint in the progression of invasive disease [70]. We showed that the IgM BCR on lymph node B cells is efficiently cleaved by rIde_*Ssuis*__h ex vivo (Figure 1).

We hypothesize that IgM B cell downregulation by Ide_*Ssuis*_ expression promotes the survival of *S. suis* in IgM-low microenvironments, such as the tonsils, as well as at sites of early invasion and infection, such as the lymph nodes, contributing to the adaptation of *S. suis* to its natural host. The long-term survival of a *S. suis* strain in the pharynx is presumably a very important factor in determining the evolutionary fitness of this strain. We speculate that Ide_*Ssuis*_ is crucial for the evolutionary fitness of *S. suis* strains because it is expressed by very different genotypes and strains of this very successful colonizer of the porcine pharynx and that Ide_*Ssuis*_ expression represents an important factor of adaptation to the pig as a natural host.

In mice and humans, IgM is secreted by B1 cells as a natural antibody and as an active response to T cell-independent antigens, such as encapsulated bacteria, and acts as an important first line of defense against various pathogens, bridging innate and adaptive immunity [29–31, 71–73]. In contrast, cps-specific IgM following infection of mice with *S. suis* cps 2 seems to be independent of the germinal center but dependent on extrafollicular T cells [74]. The importance of capsule cross-reactive IgM in activating opsonophagocytosis was further highlighted by Goyette-Desjardins et al. [75]. Dolbec et al. demonstrated that antibodies against cps following *S. suis* cps 2 infection in a mouse model were mostly of the IgM class and that depletion of IgG from the sera of infected mice did not reduce bacterial killing in an opsonophagocytosis assay [74]. Although the mouse model is well established in *S. suis* research and closely reproduces clinical signs of disease, one limitation is that mouse IgM is not cleaved by Ide_*Ssuis*_ [16, 25]. We predict that the inhibition of IgM-secreting B cells in addition to the cleavage of soluble IgM is an important mechanism by which *S. suis* evades innate immunity and the early extrafollicular IgM response to primary local invasion.

Taken together, as IgM is an important factor in the naïve host, e.g., as a natural antibody and in limiting bacterial survival, and Ide_*Ssuis*_ is not a critical virulence factor during bacteremia after experimental infection [16, 27], this leads us to assume an important role of Ide_*Ssuis*_ expression by downregulating the host immune response in the IgM-low microenvironments of colonization and early infection rather than in invasive disease. We predict that in vivo downregulation of IgM^+^ B cells and a reduction in secreted IgM levels induced by Ide_*Ssuis*_ IgM BCR cleavage provide *S. suis* with an initial survival advantage and time to establish the first steps of colonization in the host. As almost all pigs are colonized by *S. suis* [2], the expression of Ide_*Ssuis*_ may be an essential component of the adaptation of *S. suis* to its natural host.

## Supplementary Information

**Additional file 1. Antibodies and antibody dilutions used in ELISpot analysis**.

**Additional file 2. Antibodies used for ELISA**. 

**Additional file 3. Antibodies used in flow cytometry**.

**Additional file 4.  IL-2 and R848 induce B cell activation**.

## Data Availability

The datasets used and/or analysed during the current study are available from the corresponding author upon reasonable request.

## Abbreviations

BCR: B cell receptor
FCS: foetal calf serum
FMO: fluorescence minus one control
IdeE: immunoglobulin G-degrading enzyme of *Streptococcus equi* subsp. *equi*
IdeS: immunoglobulin G-degrading enzyme of *Streptococcus pyogenes*
Ide_*Ssuis*_: immunoglobulin M-degrading enzyme of *Streptococcus suis*
IdeZ: immunoglobulin G-degrading enzyme of *Streptococcus equi* subsp. *zooepidemicus*
Ig: immunoglobulin
IMDM: Iscove’s modified Dulbecco’s medium
ITAM: immunoreceptor tyrosine-based activation motif
ITIM: immunoreceptor tyrosine-based inhibitory motif
Ln: lymph node
LPS: lipopolysaccharide
MFI: median fluorescence intensity
MRP: muramidase-released protein
PBMC: peripheral blood mononuclear cells
PBS: phosphate-buffered saline
r: recombinant
RT: room temperature
SD: standard deviation
*S. suis*: *Streptococcus suis*
TLR: Toll-like receptor
