# Supplementary material for: The immunoglobulin M-degrading enzyme of Streptococcus suis (IdeSsuis) leads to long-lasting inhibition of the activation of porcine IgM-secreting B cells
Source: Vet Res. 2024 Sep 23;55:114. doi: 10.1186/s13567-024-01363-1 (PMC11421183; doi:10.1186/s13567-024-01363-1)
Supplement: Supplementary file 1 — Additional file 1. Antibodies and antibody dilutions used in ELISpot analysis. [file 13567_2024_1363_MOESM1_ESM.docx]

| **Detection of** | **specificity** | **source** | **conjugation** | **concentration or dilution** |
| --- | --- | --- | --- | --- |
| porcine IgM | anti-pig IgM  Clone K52 1C3  (Bio-Rad  # MCA637GA) | mouse | pure | 2.5 µg/mL |
| porcine IgM | anti-pig IgM  (Bethyl, # A100-117B) | goat | Biotin | 1:5000 |
| Biotin | streptavidin  (Roche Diagnostics, Mannheim, Germany, # 11089161001) |  | alkaline phosphatase | 1:2000 |
