# Supplementary material for: The immunoglobulin M-degrading enzyme of Streptococcus suis (IdeSsuis) leads to long-lasting inhibition of the activation of porcine IgM-secreting B cells
Source: Vet Res. 2024 Sep 23;55:114. doi: 10.1186/s13567-024-01363-1 (PMC11421183; doi:10.1186/s13567-024-01363-1)
Supplement: Supplementary file 2 — Additional file 2. Antibodies used for ELISA. [file 13567_2024_1363_MOESM2_ESM.docx]

| **Usage** | **specificity** | **source** | **conjugation** | **concentration or dilution** |
| --- | --- | --- | --- | --- |
| coating antibody | anti-pig IgM  Clone K521C3  (Bio-Rad, # MCA637GA) | mouse | pure | 1:500 |
| standard | swine IgM whole molecule (Rockland™, # 014-0107) | swine | pure | 1:10 000  100 ng/mL |
| secondary antibody | anti-pig IgM (Bethyl, # 100-117 B) | goat | Biotin | 1:10 000  0.1 µg/mL |
|  | Streptavidin-HRP A (R&D Systems, #DY998) |  | HRP | 1:200 |
| Detection | TMB Peroxidase substrate A (SeraCare life Sciences, #5120-0049) and TMB Peroxidase substrate B (SeraCare life Sciences, #5120-0038) |  |  |  |
