# Supplementary material for: The immunoglobulin M-degrading enzyme of Streptococcus suis (IdeSsuis) leads to long-lasting inhibition of the activation of porcine IgM-secreting B cells
Source: Vet Res. 2024 Sep 23;55:114. doi: 10.1186/s13567-024-01363-1 (PMC11421183; doi:10.1186/s13567-024-01363-1)
Supplement: Supplementary file 3 — Additional file 3. Antibodies used in flow cytometry. [file 13567_2024_1363_MOESM3_ESM.docx]

| **Name and clone** | **Antigen** | **Source and catalog** | **Conjugation** | **Concentration/**  **4 × 10^5^ cells** |
| --- | --- | --- | --- | --- |
| Mouse anti-pig IgM  Clone K521C3 | IgM Fc | Bio-Rad  # MCA637GA | pure | 0.1 – 0.15 µL |
| Purified mouse IgG1 Isotype Ctr  Clone MG1-45 | Isotype Ctr IgM Fc | Biolegend  # 401402 | pure | 0.1 – 0.15 µL |
| anti-mouse IgG1  clone RMG1-1 | Mouse IgG1 (secondary Ab for IgM Fc) | Biolegend  # 406616 | Brilliant Violet 421™ | 0.25 µL |
| mouse anti-pig CD3ε  Clone BB23-8E6-8C8 | CD3 | BD Pharmingen  # 561477 | PE-Cy™7 | 0.15 µL |
| mouse IgG2a Isotype Ctr  Clone MOPC-173 | Isotype Ctr CD3 | Biolegend  # 400231 | PE-Cy™7 | 0.15 µL |
| anti-human CD79a  Clone HM47 | CD 79a | Biolegend  # 333504 | PE | 0.5 µL |
| polyclonal rabbit anti-porcine IgM F(ab')_2_ preparation | IgM F(ab')_2_ | See Material and Methods | FITC | 0.5 µL |
| donkey anti-rabbit IgG  Clone Poly4064 | Rabbit IgG (polyclonal),  Isotype control for IgM F(ab')_2_ | Biolegend  # 406403 | FITC | 0.5 µL |
| eBioscience™Fixable viability Dye | viability | Invitrogen  # 65-0866-14 | eFluor™506 | 100 µL  1:500 diluted |
| Mouse anti-pig CD25 | CD25 | Bio-Rad  #MCA1736GA | pure | 1 µL |
